# Supplementary figures and images for: High genetic diversity of Histoplasma in the Amazon basin, 2006–2017
Source: medRxiv. 2025 Apr 3:2025.04.01.25324933. Preprint. [Version 1] doi: 10.1101/2025.04.01.25324933 (PMC11998801; doi:10.1101/2025.04.01.25324933)

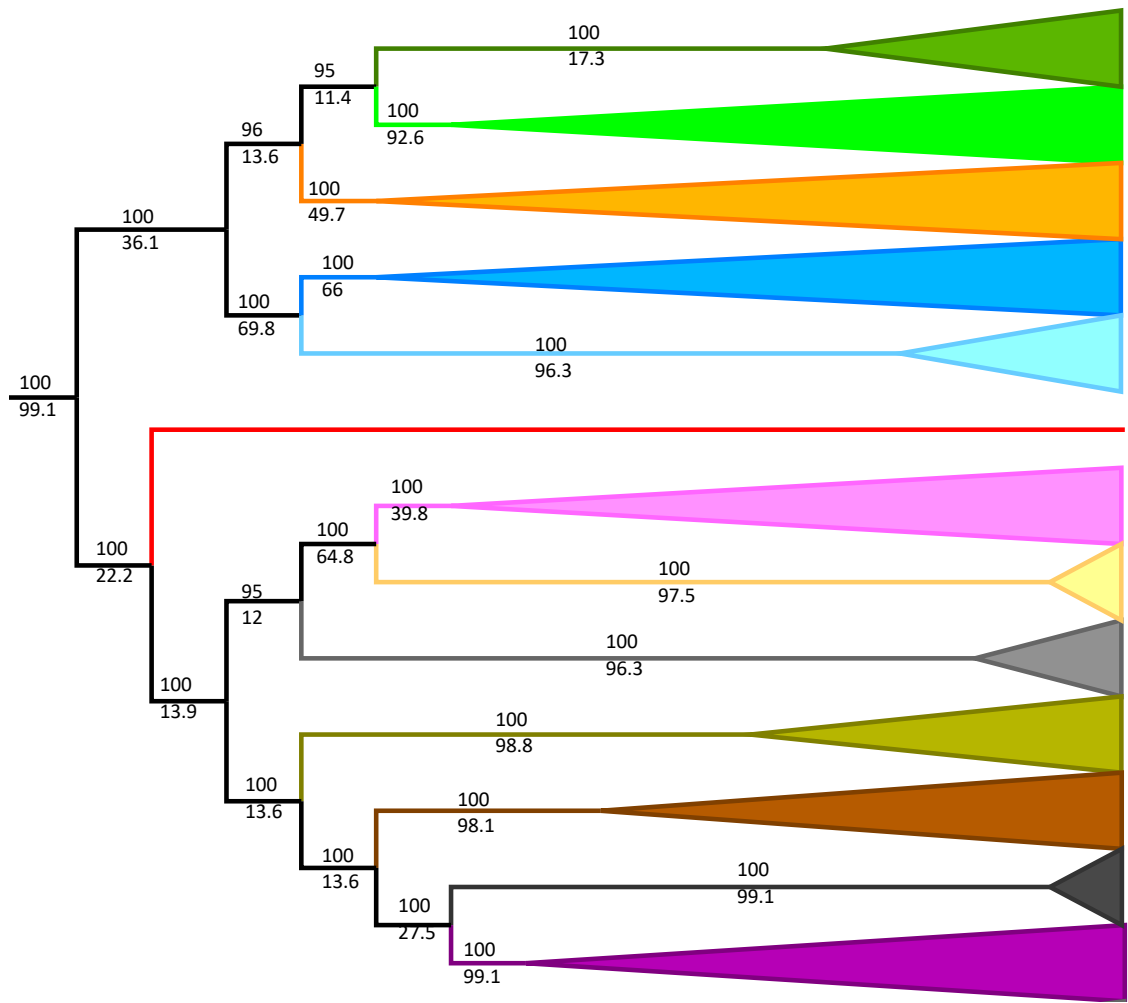

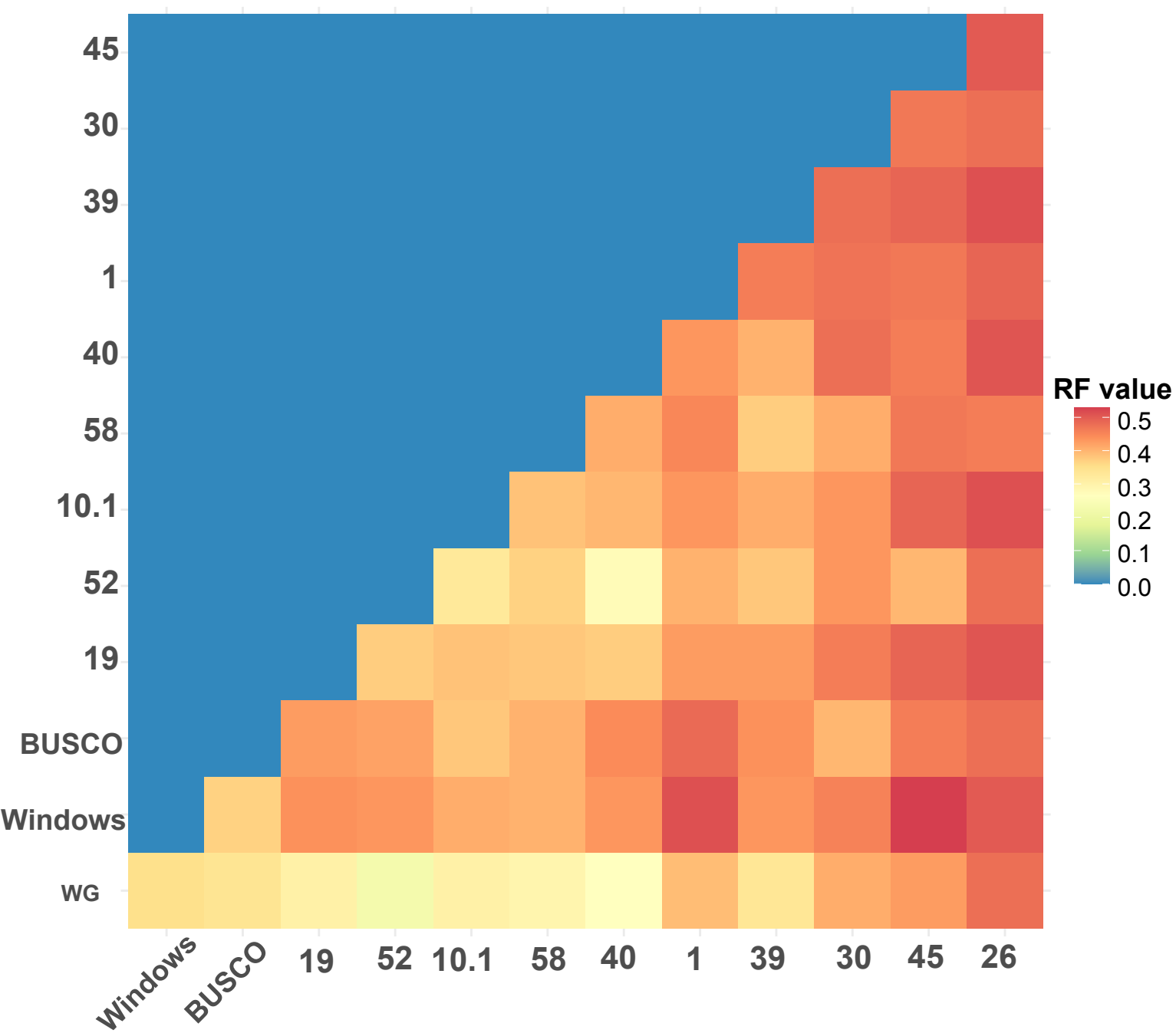

Supplement: Supplement 2 [file NIHPP2025.04.01.25324933v1-supplement-2.pdf]
